# Ulnar Compression Neuropathy Secondary to Ganglion Cyst at the Elbow Joint: A Case Report

**DOI:** 10.31729/jnma.8810

**Published:** 2024-11-30

**Authors:** Suman Kumar Basel, Prakash Shrestha, Gaurav Bir Bajracharya

**Affiliations:** 1 Department of Orthopaedic and Trauma Surgery, KIST Medical College and Teaching Hospital, Imadol, Lalitpur, Nepal

**Keywords:** *cubital tunnel*, *ganglion cyst*, *ulnar neuropathy*

## Abstract

Ulnar neuropathy is a common peripheral neuropathy, with cubital tunnel compression being the second most frequent upper extremity compression syndrome. Ganglion cysts, though rare, can contribute to ulnar nerve compression in the cubital tunnel. Here, we present a case of a 62-year-old farmer with longstanding diabetes and dyslipidemia who experienced progressive pain and numbness in his medial fingers, exacerbated by elbow flexion. Initially misdiagnosed and treated for Hansen's disease. Clinico-radiological evaluation revealed a multiloculated cystic lesion in the posteromedial aspect of his left elbow, compressing the ulnar nerve. Surgical excision of the ganglion cyst alleviated symptoms, though residual motor deficits persisted postoperatively. This case underscores the importance of considering ganglion cysts in the differential diagnosis of cubital tunnel syndrome, highlighting the diagnostic challenges and therapeutic considerations in managing this rare etiology.

## INTRODUCTION

Ulnar neuropathy is a common peripheral nerve disorder, with cubital tunnel syndrome ranking as the second most prevalent upper extremity compression syndrome following carpal tunnel syndrome. The cubital tunnel is an osseofibrous structure in the elbow. Causes of ulnar nerve compression within this tunnel include a thickened cubital tunnel retinaculum, synovial cysts, synovial chondromatosis, and notably, ganglion cysts–benign formations containing gelatinous fluid and dense connective tissue derived from synovial or tendon sheath origins.^1,2^ Here, we present a rare case of ulnar nerve compression attributable to a ganglion cyst at the elbow.

## CASE REPORT

A 62-year-old right-hand dominant farmer with diabetes and dyslipidemia presented with 18 months of pain and numbness in his medial two fingers associated with difficulty gripping objects. He also reported joint pains in his knees, wrists, and elbows for two years. No history of trauma, fever, morning stiffness, weight loss, anorexia, skin lesions, or oral ulcers. He was suspected of Hansen's disease, and received a one-month course of MDT, which was discontinued.

On examination, he had a bilateral elbow flexion deformity of 30 degrees, with further flexion to 110 degrees with painful terminal flexion and crepitus. A 3x2 cm cystic, mobile, non-tender swelling was noted over the left elbow and the overlying skin appeared normal. Clawing of the ring and little fingers, wasting of the hypothenar eminence, and first dorsal interossei on the left hand were evident, with positive Egawa, Froment, Card, and Wartenberg signs. Sensation was decreased in the little finger and ulnar half of the ring finger, with flexor digitorum profundus of these fingers graded at 3/5. Bilateral non-tender thickening of the ulnar nerve was also present.

Evaluation for an immunological disorder showed an ESR of 19 mm/hr, highly sensitive C-reactive protein at 3.14 mg/dL, and rheumatoid factor <11.4 IU/L. Leprosy was ruled out with negative slit skin smear tests and absence of aesthetic macules. The Mantoux test was negative.

**Figure 1a:**
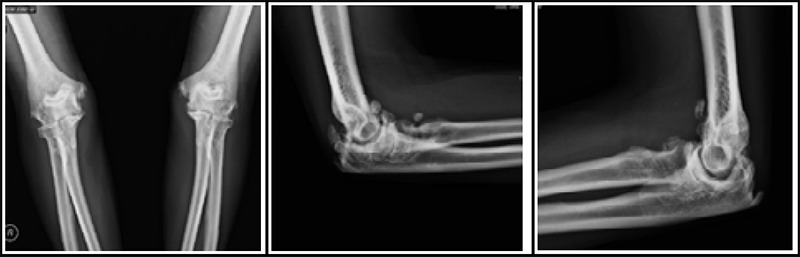
Anteriorposterior view of the right and left elbow joint, Figure 1b and 1c. Lateral view of the right and left elbow joint showing reduction in joint spaces with marginal osteophytes on the radiohumeral, ulnohumeral, and proximal radioulnar joint surfaces.

**Figure 2a and 2b:**
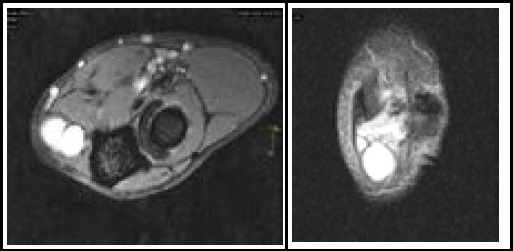
MRI of the left elbow joint showing a multiloculated cystic lesion in the posteromedial aspect with ulnar nerve stretched over its lateral border and degenerative changes. The contents of the lesion appearsT2/PD hyperintense.

A plain X-ray of the left elbow (Figure 1a-1c) revealed joint space reduction with marginal osteophytes. MRI (Figure 2a, 2b) showed a 4 × 1.5 × 1.5 cm multiloculated cystic lesion in the posteromedial elbow, with subchondral cysts near the medial epicondyle and trochlea. The lesion displaced and stretched the ulnar nerve laterally. The nerve conduction study indicated left ulnar motor sensory demyelinating neuropathy.

**Figure 3:**
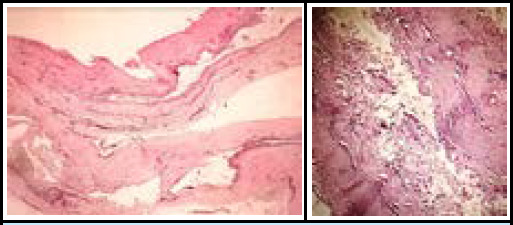
The histopathology showing cyst wall composed of fibrous tissue with few chronic inflammatory cells. Figure 3b. Cystic space lined by histiocytes and granulation tissue.

The patient underwent excision of the cyst and ulnar nerve decompression under general anesthesia. A4 × 4 cm cyst from the ulnohumeral joint, stretching the ulnar nerve, was identified and completely excised, relieving the compression. Ulnar nerve was transposed anteriorly. Postoperative recovery was uneventful, and elbow range of motion exercises started after two weeks with a Knuckle Bender Splint.

Histopathology (Figure 3a, 3b) revealed a cyst wall of fibrocollagenous tissue with a cystic space lined by histiocytes and granulation tissue. Moderate acute and chronic inflammation with prominent myxoid areas was noted, consistent with a ganglion cyst.

Regular follow-ups for six months showed some sensory improvement but no motor improvement.

## DISCUSSION

Ulnar neuropathy secondary to compression by a ganglion cyst is uncommon.^3^ Ganglion cysts, which typically occur at the end of the fifth decade of life, are usually caused by synovial herniation and tissue degeneration or repeated trauma.^2^ Unlike synovial cysts, ganglion cysts lack a synovial lining and do not communicate with the joint cavity. Ganglion cysts cause dynamic compression and stretching of the ulnar nerve, with symptoms often exacerbated by elbow flexion due to decreased cubital tunnel volume and increased pressure. The reported prevalence of ganglion cysts causing cubital tunnel syndrome is less than 8 %.^4^ Hirayuki and Tong et al. have shown associations between ganglion cysts from medial ulnohumeral joints and osteoarthritis of the elbow, with a high prevalence of osteoarthritis observed in patients with such cysts.^5,6^ In our case, radiological imaging also revealed osteoarthritic changes in the ulnohumeral and radiohumeral joints, showing an association with the ganglion cyst.

In our patient, cubital tunnel syndrome was initially diagnosed clinically and confirmed through nerve conduction studies. However, the underlying cause was identified via MRI, which suggested the presence of a ganglion cyst, later confirmed by postoperative biopsy. Identifying ganglion cysts as the cause of cubital tunnel syndrome can be challenging without MRI scans. Kato et al. reported that 33 out of 38 patients (87%) in their study had hidden ganglion cysts that went undetected during careful examination and were only discovered during surgery .^7^ Therefore, it is recommended that MRI or ultrasound be considered for patients suspected of having cubital tunnel syndrome.^6, 7^

In our patient, several risk factors contributed to ulnar neuropathy. He had metabolic conditions like Diabetes Mellitus and a history of smoking. Additionally, a bilateral elbow flexion deformity of 30° likely decreased cubital tunnel volume, as intraneural pressures rise significantly beyond 90° of elbow flexion, reducing cubital tunnel volume by 55%.^8^ Finally, his heavy manual labor as a farmer subjected his joints to stress, potentially leading to the formation of a ganglion cyst and osteoarthrosis of the elbow. These factors collectively likely contributed to the development of ulnar neuropathy.

Treatment options for cubital tunnel syndrome include in situ decompression, either by arthroscopy or open methods. Total excision of the cyst is recommended for extraneural ganglion cysts. Anterior transposition of the ulnar nerve may be performed to relieve stretching (as the nerve traverses a shorter distance) and to prevent compression by recurrence.^1^ Studies indicate that preoperative disease severity and symptom duration are predictive of postoperative motor and sensory recovery, with intrinsic muscle atrophy being the strongest correlate of poor prognosis.^9^ In our case, the patient presented with several risk factors including a prolonged history of symptoms, intrinsic muscle weakness, and sensory involvement. Additionally, the patient had a history of diabetes and smoking. Despite undergoing surgical decompression, there was notable sensory improvement observed after 6 months; however, there was no recovery of motor function, which is likely, attributable to these underlying factors.

In conclusion, this case emphasizes the complexity of diagnosing ulnar nerve compression caused by a ganglion cyst, particularly when compounded by other risk factors such as diabetes, dyslipidemia, and prolonged elbow flexion due to manual labor. Despite timely surgical intervention, residual motor deficits persisted, likely due to the chronicity of symptoms and pre-existing intrinsic muscle atrophy. This case demonstrates the critical role of advanced imaging, such as MRI, in accurately identifying atypical causes of cubital tunnel syndrome, allowing for targeted intervention and underscoring the need for early diagnosis to optimize patient outcomes.
